# Single lithium-ion channel polymer binder for stabilizing sulfur cathodes

**DOI:** 10.1093/nsr/nwz149

**Published:** 2019-10-12

**Authors:** Chaoqun Niu, Jie Liu, Tao Qian, Xiaowei Shen, Jinqiu Zhou, Chenglin Yan

**Affiliations:** College of Energy, Collaborative Innovation Center of Suzhou Nano Science and Technology, Key Laboratory of Advanced Carbon Materials and Wearable Energy Technologies of Jiangsu Province, Soochow University, Suzhou 215006, China

**Keywords:** polymer binders, single lithium-ion channels, polysulfide intermediates, lithium–sulfur batteries

## Abstract

Lithium–sulfur batteries have great potential for high-performance energy-storage devices, yet the severe diffusion of soluble polysulfide to electrolyte greatly limits their practical applications. To address the above issues, herein we design and synthesize a novel polymer binder with single lithium-ion channels allowing fast lithium-ion transport while blocking the shuttle of unnecessary polysulfide anions. *In situ* UV–vis spectroscopy measurements reveal that the prepared polymer binder has effective immobilization to polysulfide intermediates. As expected, the resultant sulfur cathode achieves an excellent specific capacity of 1310 mAh g^−1^ at 0.2 C, high Coulombic efficiency of 99.5% at 0.5 C after 100 cycles and stable cycling performance for 300 cycles at 1 C (1 C = 1675 mA g^−1^). This study reports a new avenue to assemble a polymer binder with a single lithium-ion channel for solving the serious problem of energy attenuation of lithium–sulfur batteries.

## INTRODUCTION

The growing demands on high-performance energy-storage systems for emerging technologies such as electric vehicles and artificial intelligence drive the development of high-performance batteries [1–3]. As a promising candidate of next-generation batteries, Li–S batteries have drawn much attention with their high specific capacity (1675 mAh g^−1^) and energy density (2600 Wh kg^−1^) [4,5]. However, during discharge–charge cycles, the diffusion of polysulfide in the electrolyte causes changes in the structure of the sulfur cathode; this greatly limits the commercial applications of Li–S batteries. Polymer binders, as an essential component of electrode materials, act to bond the active material and are related to the performance of batteries. Currently, various polymer binders have been developed for lithium-ion (Li-ion) batteries, including polyvinylidene fluoride (PVDF) [6,7], polytetrafluoroethylene (PTFE) [8,9] and polyacrylic acid (PAA) [10,11]. Among these, PVDF has been widely used in the rechargeable battery industry for decades due to its advantages of flame retardance and electrochemical stability [12]. Unfortunately, the conventional binder has failed to meet the requirements of emerging batteries, such as lithium–sulfur (Li–S) batteries. For example, the PVDF binder exhibits low ionic conductivity of Li ions, poor mechanical stability, and almost no inhibition on the shuttle of polysulfide; all of these factors limit the applications of Li–S batteries [13–15]. Therefore, an ideal polymer binder that overcomes the drawbacks of conventional binders is urgently needed for Li–S batteries.

A polymer with a single ion channel can restrain the transfer of unnecessary anions. This has drawn much attention to polymer electrolyte because of the reduction of concentration polarization caused by transferring anions and lower electrolyte loss [16]. Several strategies have been developed to improve the properties of the polymer electrolyte, such as organic polymer [17,18] and organic–inorganic hybrid polymers [19]. It is a fact that single Li-ion channels can efficiently improve Li-ion transfer and reduce internal polarization [16]. In this regard, it would be a significant effort to employ polymer binders with single Li-ion channels in Li–S batteries to inhibit the transport of polysulfide and stabilize the Li-ion conductivity.

In this work, we report a novel polymer binder with a single Li-ion channel via the formation of selective transport channels by aliphatic sulfur to stabilize the cycle performance of sulfur cathode. The designed polymer contains a hard phase and a soft phase to obtain excellent fracture strain and promising tensile stress, which is beneficial to reduce the volume change of electrode materials [20]. The resultant polymer binder is demonstrated to effectively inhibit the shuttle effect of polysulfide intermediates due to the immobilization of polysulfide ions by single Li-ion channels. The designed sulfur cathodes achieve an improved specific capacity of 1310 mAh g^−1^ at 0.2 C and enhanced cycling performance. Promising physical structure retention of the prepared sulfur cathode and low consumption of the Li metal anode are observed.

## RESULTS AND DISCUSSION

**Figure 1.**
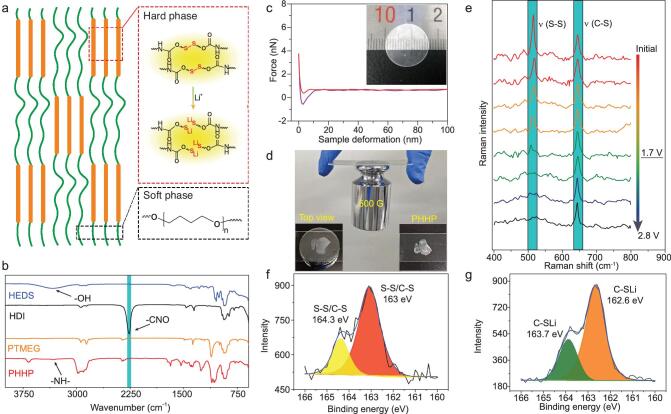
Synthesis and characterization of polymer binders. (a) Schematic diagrams of the formation of the polymer with the single ion channels. (b) The FTIR of HEDS, HDI, PTMEG, and PHHP. (c) The typical force–displacement curve of PHHP film. Inset is the optical image of PHHP film. (d) Adhesion experiments of the binder. (e) In operando Raman spectroscopy of the PHHP electrode. The S 2p XPS spectra of PHHP electrode (f) before discharge and (g) after a discharge–charge cycle.

Figure 1a shows a schematic diagram of polymer binder (PHHP) prepared by the polymerization of bis(2-hydroxyethyldisulfide) (HEDS), hexamethylene diisocyanate (HMDI) and polytetramethylene ether glycol (PTMEG). The synthetic routes for the PHHP binder are shown in Fig. S1 (online supporting information). Further, in order to remain the mechanical structure after discharge–charge cycles, the designed polymer binder consists of stable soft and hard segments featuring strong bonding interactions and dynamic aliphatic disulfides. The PTMEG is used as the soft segment in the polymer, which contributes to the elasticity of the polymer. The HMDI and HEDS are used as the hard segment, enhancing the strong interaction of amide groups. The hard phase of the polymer provides a stable framework and the soft phase contributes to the elasticity. The volume changes and cracking of sulfur cathode are expected to improve by the comprehensive effect of the hard phase and the soft phase.

**Figure 2.**
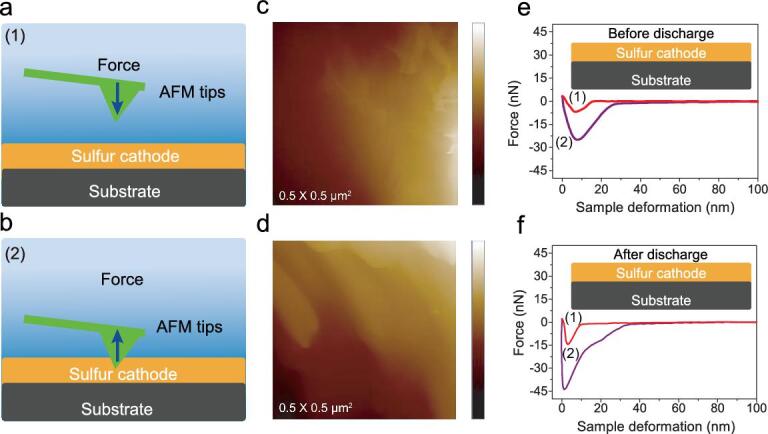
AFM measurements of initial sulfur cathode and discharged sulfur cathode with PHHP binder. The schematic diagrams of (a) loading process and (b) unloading process. AFM images for (c) initial PHHP electrode, color scale, 0–200 nm and (d) discharged PHHP electrode, color scale, 0–250 nm. Typical force–displacement curve of (e) initial sulfur cathode and (f) discharged sulfur cathode with PHHP binder.

Fourier-transform infrared spectra (FTIR) and Raman spectra of HEDS, HMDI, PTMEG, and PHHP are shown in Figs 1b and S2, respectively. The disappearance of the isocyanate group (–CNO) (2259 cm^−1^) and hydroxyl group (3345 cm^−1^) vibration and the appearance of a tertiary amino group (3320 cm^−1^) vibration in spectra of PHHP confirm the polymerization. The peaks of the PHHP binder skeleton (–S–S–, 510 cm^−1^; –C–S–, 640 cm^−1^; –C–NH–, 1125 cm^−1^) are also clearly measured as shown in Fig. S2 [21]. As shown in Fig. 1c, the Young’s modulus of PHHP film is 250 MPa tested by atomic force microscopy (AFM) and an image of the PHHP film is shown in Fig. S3. The inset shows the uniform and transparent PHHP film prepared by casting the precursor resins on Teflon plates, revealing the strong mechanical strength and excellent binding performance of PHHP support for a 500 g weight (Fig. 1d). The adhesion experiment clearly demonstrates a promising practical application of PHHP for a polymer binder.

In order to understand the reaction between the polymer binder and Li ion, an electrode containing the PHHP and conductive carbon was prepared for the discharging–charging tests. During discharging, the disulfide groups in the polymer fracture, combine with Li ions and assemble into a fast channel for Li-ion transfer. Figure 1e demonstrates *in situ* Raman spectra of the PHHP electrode during the initial discharging–charging process at 10 μA cm^−2^ [22]. During the discharging of cells, the Raman intensities of –S–S– peaks decrease gradually due to the formation of aliphatic Li sulfide. During the subsequent charging process, the S–S peak does not reappear, indicating the irreversible S–S bond cleavage reaction, which favors the stability of the binder. There are no obvious Raman intensity changes of –C–S– peaks. The discharge–charge curves of the PHHP electrode are shown in Fig. S4. Further, X-ray photoelectron spectroscopy (XPS) spectra of the initial PHHP electrode and the discharged PHHP electrode are shown in Fig. 1f and 1g, respectively. For the S 2p spectra of the initial sample, two characteristic peaks of S–S/S–C (164.3 eV and 163 eV) are detected as shown in Fig. 1f. After discharging of the PHHP electrode, S 2p XPS spectra show that the S–S/C–S peaks disappear while two other characteristic peaks of C–SLi (163.7 eV and 162.6 eV) are detected as illustrated in Fig. 1g. The results of S 2p XPS spectra are consistent with *in situ* Raman spectroscopy analysis. As shown in Fig. S5, the C 1s, N 1s, and O 1s XPS spectra are measured and the C–C, C–O, and C–N groups (284.5, 286.5 and 399 eV) before and after the cycles demonstrate the stability of the PHHP binder.

AFM was used to investigate the mechanical stability of sulfur cathodes with PHHP binder before and after cycling. The topographic images of Fig. 2c and 2d exhibit uniform surfaces of sulfur cathode before and after discharge, respectively. Schematic diagrams of the loading process (1) and the unloading process (2) during the measurement are clearly shown in Fig. 2a and 2b. Figure 2e and 2f display the force calculated by subtracting the cantilever defect distance from the total piezoelectric actuator as a function of sample deformation and the insets are schematic diagrams of the measured samples before and after the cycles [23,24]. The intersection curve and the independent position curve of the approach to the extrapolation line are set to zero indented positions. The slope of the loading curve of sulfur cathode before discharge is lower than that after discharge, which demonstrates the excellent viscoelasticity of the PHHP binder. According to Johnson–Kendall–Roberts (JKR) models, the increase in modulus could be estimated by fitting the unloading curves [25]. From the JKR model fit, sulfur cathodes before and after discharge (Fig. 2d) show moduli of 0.7 GPa and 1.0 GPa, respectively. So, the sulfur cathode with a single ion channel shows a more stable mechanical property than the initial sample. The improvement of cathode modulus is mainly due to the reinforcing effect by the generation of single Li-ion channels in PHHP, and those channels are inorganic forged C–SLi groups. Thus, the cathode modulus is improved after discharge. In contrast, sulfur with PVDF binder was measured by AFM. Topographic images and typical force–displacement curves before and after discharge are shown in Fig. S6. After the first discharge, the surface of the sulfur cathode became more uneven (Fig. S6c) than the initial images (Fig. S6a), obviously. Then, the modulus of the sulfur cathode decreased from 610 MPa (Fig. S6b) to 520 MPa (Fig. S6d) after discharge, which was due to the PVDF being unable to retain its mechanical structural stability during electrode reactions.

**Figure 3.**
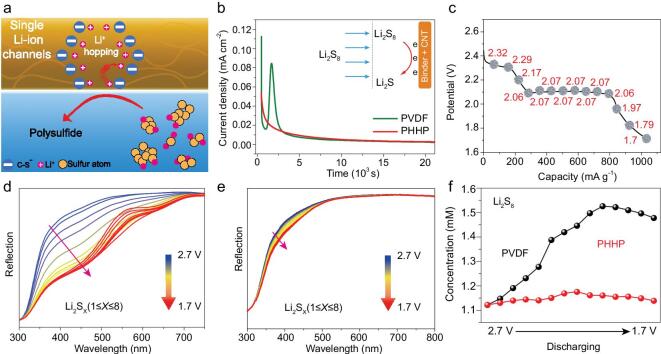
(a) Schematic diagrams of single ion channels regulate the polysulfide and Li–ion hopping. (b) Potentiostatic discharge curves of PDVF and PHHP electrodes with the same weight at 2.08 V for Li_2_S deposition. (c) The discharge curve of *in situ* UV battery. *In situ* UV–vis spectra of assembly Li–S cells with (d) PVDF binder and (e) PHHP binder as discharge at 0.2 C. (f) Li_2_S_8_ at different potentials in Li–S batteries with PHHP binder (red curve) and PVDF binder (black curve).

**Figure 4.**
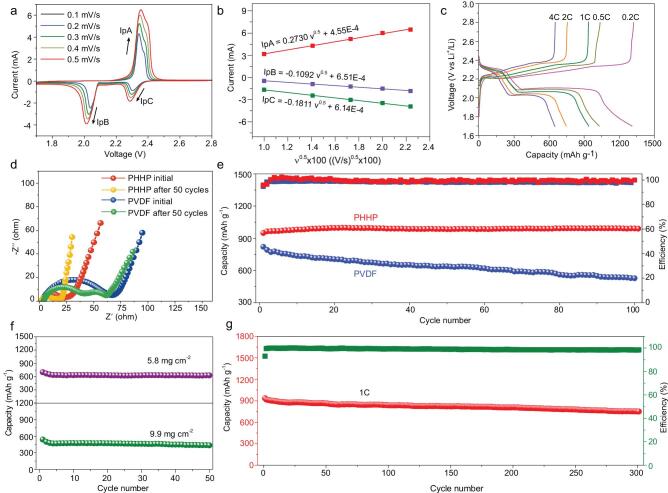
Electrochemical performance of cells with PHHP binder. (a) Representative voltammograms of the sulfur cathode with PHHP binder obtained at different scan rates. (b) Linear relationship of I_p_ and v^0.5^. (c) Discharge–charge curves at the different current density of Li–S cells with PHHP. (d) Nyquist plots of Li–S cells with PHHP before and after 50 cycles and with PVDF before and after 50 cycles. (e) Cycling performance and Coulombic efficiency of Li–S cells at 1 C for 100 cycles. (f) Cycling performance of high loading sulfur cathode with PHHP of 5.8 mg cm^−2^ and 9.9 mg cm^−2^ at the current density of 0.5 mA cm^−2^. (g) Discharge capacity and Coulomic efficiency of Li–S cells with PHHP at 1 C for 300 cycles.

The single Li-ion channel of the binder regulates the polysulfide intermediates and contributes to the Li-ion conductivity as shown in the schematic diagram (Fig. 3a). According to the Donnan exclusion principle, a single ion channel can regulate the polysulfide [26]. In order to confirm the barrier effect of a single Li-ion conducting channel on polysulfide intermediates, the kinetics of the Li_2_S nucleation experiment was measured by monitoring the current flow caused by polysulfide intermediate deposition [27–29]. Coin cells (2025) with PHHP- and PVDF-based electrodes as the cathode and Li metal as the anode were measured using an electrochemical workstation (CHI660E). A Li_2_S_8_ solution prepared by dissolving 20 mM Li_2_S_8_ in 1, 3-dioxolane and 1, 2-dimethoxyethane with equal volume concentration was used as the catholyte and conventional Li–S battery electrolyte was used as the anolyte, respectively. The affinities of PHHP and PVDF to polysulfide are linked to the current flow under the constant driving force of sulfur species reduction at 2.08 V. As shown in Fig. 3b, the drop in current at the beginning corresponds to the transport of Li_2_S to the cathode under 2.08 V. Subsequently, the obvious current peaks of the PVDF-based electrode were measured; these are due to Li_2_S_8_ in electrolyte deposited on PVDF-based electrode by large amounts of reduction reactions. Further, the strong adsorption of polysulfide by PVDF leads to the significant deposition of Li_2_S. In contrast, such considerable peak intensity cannot be obtained in discharged PHHP binder, which indicates that the PHHP electrode’s weak adsorption of polysulfide as PVDF avoids the shuttle effect of polysulfide, thereby contributing to the great retention of capacity for Li–S batteries. Furthermore, *in situ* ultraviolet and visible (UV–vis) spectroscopy was measured to investigate the component conversion in the electrolyte of Li–S batteries according to the principle of Beer–Lambert’s law [30,31]. As the polysulfide dissolves during the discharge and then enters the electrolyte, the UV–vis reflection signal will get weaken. The same weight sulfur cathode with PVDF binder and PHHP binder was assembled into well designed cells for *in situ* UV measurements. The discharge curve is shown in Fig. 3c. In Fig. 3d, the measured UV–vis spectra of the sulfur cathode with PVDF shows a continuous decrease of reflection in the range of 350–600 nm, linked to the concentration changes of polysulfide. The concentration change of polysulfide in the electrolyte was derived from the diffusion of polysulfide through the glass fiber separator in the voltage range from 2.7–1.7 V. A slight decrease of reflection compared to sulfur cathode with PVDF in sulfur cathode with PHHP was observed (Fig. 3e). The concentration values of different polysulfides can be calculated via the relationship between the concentration and actual UV intensity, according to the previous report on different wavelengths for polysulfides (λ = 450 nm corresponding to Li_2_S_2_, λ = 505 nm to Li_2_S_4_, λ = 530 nm to Li_2_S_6_ and λ = 560 nm to Li_2_S_8_) [32]. Figure 3f shows that the concentration of Li_2_S_8_ in both cells increases with discharge. However, the concentration of Li_2_S_8_ in sulfur cathode with PVDF reached a high level. As a comparison, a low level was observed in sulfur cathode with PHHP. Figure S7 displays the dissolution of polysulfide in conventional Li–S electrolyte at a discharging current density of 0.5 C in Li–S cells with PVDF binder and PHHP binder, respectively. During the discharge, the electrolyte in Li–S cells with PVDF changes from colorless to yellow over 3 h, due to the dissolution of polysulfide. Conversely, the electrolyte in Li–S cells with PHHP binder remains colorless in the same conditions. This apparent contrast reveals that the PHHP binder has a great effect on the cycle stability of Li–S batteries. Thus, the single Li-ion conducting channel formed by PHHP in sulfur cathode has a considerable inhibition on the shuttle effect of polysulfide.

To demonstrate the performance of the polymer binder in the transportation of lithium ions, the Li-ion diffusion coefficients (*D*_Li_) of sulfur cathode with PHHP binder and PVDF binder after 10 cycles of activation treatment were evaluated using the Randles–Sevcik equation, as below:

(1) \begin{equation*} {I}_{\mathrm{p}}=2.69\times {10}^5{n}^{1.5}A{D}_{\mathrm{Li}}^{0.5}{v}^{0.5}{C}_{\mathrm{Li}} \end{equation*}

where ${I}_{\mathrm{p}}$ is the peak current, *n* indicates the number of electrons in the reaction (*n* = 2 in Li–S batteries), *A* is the effective electrode area (*A* = 1.13 cm^2^), *v* is the voltage scanning rate and *C*_Li_ corresponds to the Li-ion concentration in the electrolyte (*C*_Li_ = 1.16 × 10^−3^ mol cm^−3^) [33,34]. As shown in Fig. 4a, representative voltammograms of the sulfur cathode with PHHP binder are obtained from 0.1–0.5 mV s^−1^. The linear relationships of *I*_p_ and *v*^0.5^ (*I*_p_A, cathodic peak at ∼2.40 V; *I*_p_B, cathodic peak at ∼2.05 V and I_p_C, cathodic peak at ∼2.30 V) are shown in Fig. 4b. For the sulfur cathode with PHHP binder, *D*_Li_A = 7.49 × 10^−8^, *D*_Li_B = 1.20 × 10^−8^, *D*_Li_C = 3.30 × 10^−8^ are confirmed. As a comparison, the representative voltammograms of the sulfur cathode with PVDF binder from 0.1–0.5 mV s^−1^ and its linear relationship of *I*_p_ and *v*^0.5^ are shown in Fig. S8. For the sulfur cathode with PHHP binder, *D*_Li_A = 4.36 × 10^−8^, *D*_Li_B = 1.04 × 10^−8^, *D*_Li_C = 2.09 × 10^−8^ are confirmed. The improved *D*_Li_ of sulfur cathode is due to the fast Li-ion transport in PHHP binder. This suggests that the polymer binder with a single Li-ion channel allows fast Li-ion transport. The electrochemical performance of resultant sulfur cathode was also assessed. As shown in Fig. S9a, the typical CV profiles for sulfur cathode with PHHP were measured in the range of 1.7 V and 2.8 V at a scan rate of 0.1 mV s^−1^. Two reduction peaks (2.29 V, 2.02 V) were detected, corresponding to the conversion from sulfur to long-chain lithium polysulfide (Li_2_S*_x_*, 4 ≤ *x* ≤ 8) and further conversion to short-chain lithium polysulfide (Li_2_S*_x_*, 1 ≤ *x* ≤ 4), respectively. Relatively, two oxidation peaks correspond to the transition from Li_2_S/Li_2_S_2_ to Li_2_S_3_/Li_2_S_4_ and then oxidation to sulfur. In the initial cycles, the oxidation peaks display an obvious overpotential of cathodic peaks and the reason for this is that the sulfur rearranges itself and transfers to electrochemically favorable positions [35,36]. In Fig. S9a, the overpotential of oxidation peaks gradually disappears, as the number of discharge–charge cycles increases. The positive shift of reduction peaks in CV profiles after the first sweep demonstrates the enhanced kinetics of polysulfide intermediate redox. The stable CV profiles reveal highly reversible redox reactions and cycling stability in Li–S batteries. As shown in Fig. S9b, the rate discharge capacities of sulfur cathode with PHHP are 1310 (0.2 C), 1020 (0.5 C), 950 (1 C), 750 (2 C) and 650 mAh g^−1^ (4 C), which are all higher than the discharge capacities of sulfur cathode with PVDF, respectively. Stable discharge–charge curves of different current densities for sulfur cathode with PHHP are shown in Fig. 4c. Discharge and charge plateaus are clearly observed, which are consistent with the CV curves. The formation of a stable single Li-ion conducting channel contributes to Li-ion conductivity and avoids the shuttle effect of polysulfide.

**Figure 5.**
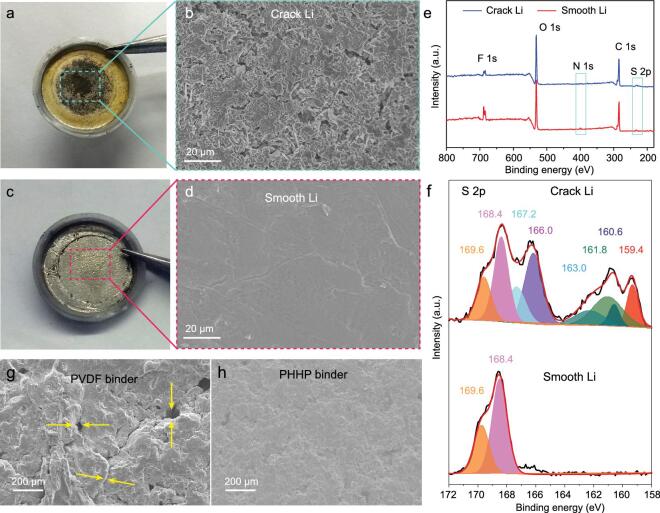
(a) The optical images and (b) SEM image of Li metal in PVDF binder cell after 50 cycles at 0.5 C. (c) The optical images and (d) SEM image of Li metal in PHHP binder cell after 50 cycles at 0.5 C. (e) The XPS spectrum and (f) S 2p spectra of crack Li metal and smooth Li metal. (g) The SEM image of sulfur cathode in PVDF binder cell and (h) SEM image of sulfur cathode in PHHP binder cell after 50 cycles at 0.5 C.

Electrochemical impedance spectroscopy (EIS) tests of sulfur cathode with PHHP and sulfur cathode with PVDF before and after 50 cycles were measured as shown in Fig. 4d. The interface resistance of the batteries links to the radius value of a semicircle in the high-frequency region. The interface resistance of the initial sulfur cathode with PHHP (about 25 Ω) is obviously lower than the resistance of the initial sulfur cathode with PVDF (about 70 Ω), which indicates the excellent interface property of sulfur cathode with PHHP. After 50 cycles, the sulfur cathode with PHHP cells exhibits a much lower interface resistance value in the high-frequency region (20 Ω) than the sulfur cathode with PVDF cells (65 Ω). This is attributed to the stable PHHP/electrolyte interface and the inhibitory deposition of Li_2_S/Li_2_S_2_ on the Li metal surface. A comparison of cycling performance between the sulfur cathode with PHHP cells and sulfur cathode with PVDF cells at a current density of 0.5 C for 100 cycles is shown in Fig. 4e. The sulfur cathode with PHHP cells exhibits a high initial discharge capacity of 950 mAh g^−1^, and the discharge capacity retention after 100 cycles was 98%. In contrast, the sulfur cathode with PVDF cells delivers a low initial discharge capacity (800 mAh g^−1^) and a fast decline of capacity, decreasing from 800 mAh g^−1^ to 530 mAh g^−1^. The average Coulombic efficiency of sulfur cathode with PHHP (99.5%) is higher than that of the sulfur cathode with PVDF (95.2%). Note that the high Coulombic efficiency was due to the immobilized shuttle effect of polysulfide and the excellent Li-ion conductivity in PHHP binder. The high loading of active materials is an important issue to be considered in actual production. Also, the sulfur cathode with PHHP cells retains a promising cycling performance after 50 cycles even with the loading of 5.8 mg cm^−2^ and 9.9 mg cm^−2^ as shown in Fig. 4f. To measure the long cycling stability, the long-term cycling performance of sulfur cathode with PHHP at a current rate of 1 C is presented in Fig. 4g. The capacity retention of 85% after 300 cycles and the Coulombic efficiency can remain above 98.5% after discharge–charge cycling. The electrochemical performance reveals the single Li-ion conducting channel formed by the PHHP binder contribution to the stable discharge–charge cycling performance and high capacity retention.

During cycling of Li–S batteries, the dissolved polysulfide from sulfur cathode can deposit on the Li metal. The deposited sulfide can accelerate the lithium dendrite growth and reduce the Coulombic efficiency of the lithium metal. To analyze the shuttle effect of polysulfide, optical scanning electron microscopy (SEM) images and XPS spectra of Li metal after 50 cycles at 0.5 C in different polymer binder cells were measured. Figure 5a shows that the middle of the Li metal is black and surrounded by yellow in PVDF binder cells, due to the severe polysulfide shuttling effect and Li dendrite growth accelerated by polysulfide deposition, and the SEM image in Fig. 5b shows the violent destruction of Li metal. The effective inhibition of the polysulfide shuttling effect reduces the deposition of polysulfide, thus slowing down the loss of active Li metal. As an obvious comparison, the metallic surface of the Li sheet in PHHP binder cells is shown in Fig. 5c. Figure 5d shows a smooth Li surface. The XPS spectra of cracked Li and smooth Li were measured to confirm the deposition of sulfide. As shown in Fig. 5e, the S 2p, F 1s, N 1s, F 1s, C 1s and O 1s peaks were all observed from the XPS spectrum of cracked and smooth Li metal. The S 2p spectra are shown in Fig. 5f. From the spectra of cracked Li metal, the main components are the sulfate (169.6 and 168.4 eV doublet), thiosulfate (167.2 and 166.0 eV doublet), Li_2_S_2_ (163.0 and 161.8 eV doublet) and Li_2_S (160.6 and 159.4 eV doublet), which are from the decomposition of the electrolyte and the reaction between soluble polysulfide and Li metal. The S 2p spectra of smooth Li metal do not observe the obvious peaks of Li_2_S_2_ and Li_2_S, which demonstrate that almost no polysulfide is deposited on the Li metal. The SEM image of the sulfur cathode with PVDF binder after 50 discharge–charge cycles at 0.5 C was measured to evaluate the damage to the cathode piece. Figures 5g and S10a shows severe cracks observed on the surface of sulfur cathode with PVDF binder, which are responsible for capacity loss and the low Coulomb efficiency of Li–S batteries. As an obvious comparison, Figs 5h and S10b show sulfur cathodes with PHHP binder showing surface uniform structure. The slight damage to the sulfur cathode is due to the mechanical stability of PHHP binder. In discharge–charge cycling, the sulfur and polysulfide intermediates are trapped inside the single Li-ion conducting channels. The effectively shuttle of Li-ions can greatly protect the stability of the cycle and the integrity of the sulfur electrode. All of the above research data show that the Li–S battery with PHHP binder exhibits excellent cycle performance and the formed single Li-ion conducting channel of PHHP binder is an effective strategy to inhibit the shuttle effect of polysulfide intermediates for improved performance of Li–S batteries.

## CONCLUSIONS

In summary, we have successfully prepared a novel polymer binder with a single Li-ion channel to develop high-performance sulfur cathodes. The *in situ* Raman measurements demonstrate the formation of the single Li-ion channel, which regulates the transfer of unnecessary polysulfide anions and contributes to Li-ion hopping. The polymer binder is confirmed to effectively immobilize the shuttle effect of polysulfide intermediates by the *in situ* UV–vis measurement. The resultant sulfur cathode achieves an excellent specific capacity of 1310 mAh g^−1^ at 0.2 C, high Coulombic efficiency (99.5% at 0.5 C after 100 cycles) and stable cycling performance (capacity retention of 85% after 300 cycles at 1 C). Moreover, the excellent adhesion and mechanical stability of PHHP binder maintain the structural integrity of sulfur cathode after discharge–charge cycles. These results demonstrate the promising improvement of Li–S batteries by the PHHP binder and we believe that the reported polymer binder with single Li–ion channels is one of the most effective strategies for high-energy Li–S batteries.

## METHODS

### Preparation of polymer binder

Polytetramethylene ether glycol (PTMEG) was first heated at 110°C under vacuum for 3 h to remove the moisture and then mixed evenly with bis(2-hydroxyethyldisulfide) (HEDS) and hexamethylene diisocyanate (HMDI) with a molar ratio of 1:1:2. Next, dibutyltin dilaurate (DL) as a catalyst was added to the precursor solution to complete the polymerization at 70°C for 12 h. Then the polymer binder by the polymerization of HEDS, HMDI and PTMEG (PHHP) was prepared and all reaction processes were evenly stirred.

### Material characterization

To confirm the occurrence of polymerization, the PTMEG, HEDS, HDMI, and PHHP were characterized using a Fourier-transform infrared spectrometer (FTIR, Bruker) in the range 4000–400 cm^−1^, a Raman spectrometer (HR Evolution) in the range 3300–400 cm^−1^ and X-ray photoelectron spectroscopy (XPS). The morphology was observed by field emission scanning electron microscopy (FESEM, SU8010, Japan). Atomic force microscopy (AFM) measurements were taken using a Bruker Dimension Icon with a Nanoscope V controller.

### Electrochemical measurement

The sulfur/carbon nanotube (S/C) active materials were prepared by mixing sulfur (Alfa Aesar, AR) and highly purified single-walled carbon nanotube with special surface area in the range 500–700 m^2^ g^−1^ (Nanjing Xianfeng Nano Material Technology Co., Ltd, purity >95%, diameter in the range 1–2 nm) (7:3 in mass), and then ball milled for 10 h. The mixture was heated at 160°C under argon gas atmosphere. Then active materials (90 wt%) and PHHP (10 wt%) were fully dispersed in NMP to form a homogeneous slurry. The mixed slurry was uniformly coated on an Al-foil current collector using a doctor blade method and dried at 60°C for 24 h under vacuum to remove the solvent and then punched into 1.13 cm^2^ discs. The sulfur cathodes were prepared with a mass loading of 2.0 mg cm^−2^ and the sulfur content was about 63 wt%. The PHHP binder-based Li–S batteries performed in 2025 coin cells with 25 μL conventional Li–S liquid electrolyte (1 M lithium bis(trifluoromethanesulfonyl)imide (LiTFSI) in 1,3-dioxolane/1,2-dimethoxyethane (DOL/DME), DodoChem). The discharge–charge property and cyclic voltammetry test were measured using a CT2001A cell test instrument (Wuhan LAND Electronic Co. Ltd) and CHI660E (Shanghai Chenhua Instrument Co. Ltd) electrochemical workstation, respectively.

To investigate the shuttle effect of Li_2_S*_x_* (2 ≤ *x* ≤ 8) in Li–S batteries, a special battery was assembled using a tailor-made coin cell case with a glass window for UV–vis spectroscopy (UV Lambda 750S). To ensure that the electrolyte could be exposed in UV light, the Li anode shell needed to be punched before assembly. The galvanostatic discharge–charge cycling was carried out with a CHI660E (Shanghai Chenhua Instrument Co. Ltd) electrochemical workstation at a current density of 0.8375 mA cm^−2^ (mass loading of sulfur ≈ 1.0 mg). The UV–vis spectra were measured at different potentials of coin cells in the range 800 nm—300 nm with a sampling interval of 0.5 nm.

## Supplementary Material

nwz149_Supplemental_File

## References

[1] Armand M , Tarascon JM. Building better batteries. Nature 2008; 451: 652–7. 18256660 10.1038/451652a

[2] Goodenough JB , Kim Y. Challenges for rechargeable Li batteries. Chem Mater 2009; 22: 587–603.

[3] Van Noorden R . The rechargeable revolution: a better battery. Nature 2014; 507: 26–8. 24598624 10.1038/507026a

[4] Bruce PG , Freunberger SA, Hardwick LJ et al. Li–O_2_ and Li–S batteries with high energy storage. Nat Mater 2012; 11: 19–29. 10.1038/nmat3191 22169914

[5] Manthiram A , Fu Y, Su YS. Challenges and prospects of lithium–sulfur batteries. Acc Chem Res 2012; 46: 1125–34. 23095063 10.1021/ar300179v

[6] Markevich E , Salitra G, Aurbach D. Influence of the PVDF binder on the stability of LiCoO_2_ electrodes. Electrochem Commun 2005; 7: 1298–304.

[7] Zhao J , Yang X, Yao Y et al. Moving to aqueous binder: a valid approach to achieving high-rate capability and long-term durability for sodium-ion battery. Adv Sci 2018; 5: 1700768. 10.1002/advs.201700768 PMC5908374 29721423

[8] Kim T , Jung G, Yoo S et al. Activated graphene-based carbons as supercapacitor electrodes with macro- and mesopores. ACS Nano 2013; 7: 6899–905. 23829569 10.1021/nn402077v

[9] Wu ZS , Wang DW, Ren W et al. Anchoring hydrous RuO_2_ on graphene sheets for high-performance electrochemical capacitors. Adv Funct Mater 2010; 20: 3595–602.

[10] Koo B , Kim H, Cho Y et al. A highly cross-linked polymeric binder for high-performance silicon negative electrodes in lithium ion batteries. Angew Chem Int Ed 2012; 51: 8892–7. 10.1002/anie.201201568 22847982

[11] Kim Y , Park Y, Choi A et al. An amorphous red phosphorus/carbon composite as a promising anode material for sodium ion batteries. Adv Mater 2013; 25: 3045–9. 23494991 10.1002/adma.201204877

[12] Chen H , Ling M, Hencz L et al. Exploring chemical, mechanical, and electrical functionalities of binders for advanced energy-storage devices. Chem Rev 2018; 118: 8936–82. 30133259 10.1021/acs.chemrev.8b00241

[13] Pang Q , Liang X, Kwok CY et al. Advances in lithium–sulfur batteries based on multifunctional cathodes and electrolytes. Nat Energy 2016; 1: 16132.

[14] Xu R , Lu J, Amine K. Progress in mechanistic understanding and characterization techniques of Li-S batteries. Adv Energy Mater 2015; 5: 1500408.

[15] Chen Y , Lu S, Zhou J et al. Synergistically assembled Li_2_S/FWNTs@ reduced graphene oxide nanobundle forest for free-standing high-performance Li_2_S cathodes. Adv Funct Mater 2017; 27: 1700987.

[16] Feng Y , Tan R, Zhao Y et al. Insight into fast ion migration kinetics of a new hybrid single Li-ion conductor based on aluminate complexes for solid-state Li-ion batteries. Nanoscale 2018; 10: 5975–84. 29542770 10.1039/c8nr00573g

[17] Ma Q , Zhang H, Zhou C et al. Single lithium-ion conducting polymer electrolytes based on a super-delocalized polyanion. Angew Chem Int Ed 2016; 55: 2521–5. 10.1002/anie.201509299 26840215

[18] Amaresh S , Karthikeyan K, Kim KJ et al. Aluminum based sulfide solid lithium ionic conductors for all solid state batteries. Nanoscale 2014; 6: 6661–7. 24816684 10.1039/c4nr00804a

[19] Lago N , Garcia Calvo O, Lopez del Amo JM et al. All-solid-state lithium-ion batteries with grafted ceramic nanoparticles dispersed in solid polymer electrolytes. ChemSusChem 2015; 8: 3039–43. 26373359 10.1002/cssc.201500783

[20] Lai Y , Kuang X, Zhu P et al. Colorless, transparent robust, and fast scratch-self-healing elastomers via a phase-locked dynamic bonds design. Adv Mater 2018; 20: 1802556. 10.1002/adma.201802556 30073707

[21] Parnell S , Min K, Cakmak M et al. Kinetic studies of polyurethane polymerization with Raman spectroscopy. Polymer 2003; 44: 5137–44.

[22] Chen JJ , Yuan RM, Feng JM et al. Conductive Lewis base matrix to recover the missing link of Li_2_S_8_ during the sulfur redox cycle in Li–S battery. Chem Mater 2015; 27: 2048–55.

[23] Li G , Gao Y, He X et al. Organosulfide-plasticized solid–electrolyte interphase layer enables stable lithium metal anodes for long-cycle lithium–sulfur batteries. Nat Commun 2017; 8: 850. 29021575 10.1038/s41467-017-00974-x PMC5636837

[24] Gu Y , Wang WW, Li YJ et al. Designable ultra-smooth ultra-thin solid–electrolyte interphases of three alkali metal anodes. Nat Commun 2018; 9: 1339. 29632301 10.1038/s41467-018-03466-8 PMC5890267

[25] Pharr G , Oliver W. Measurement of thin film mechanical properties using nanoindentation. MRS Bull 1992; 17: 28–33.

[26] Lee J , Song J, Noh H et al. A nanophase-separated, quasi-solid-state polymeric single-ion conductor: polysulfide exclusion for lithium–sulfur batteries. ACS Energy Lett 2017; 2: 1232–9.

[27] Fan FY , Carter WC, Chiang YM. Mechanism and kinetics of Li_2_S precipitation in lithium–sulfur batteries. Adv Mater 2015; 28: 5203–9. 10.1002/adma.201501559 26257297

[28] Chen CY , Peng HJ, Hou TZ et al. A quinonoid-imine-enriched nanostructured polymer mediator for lithium–sulfur batteries. Adv Mater 2017; 29: 1606802. 10.1002/adma.201606802 28417502

[29] Liu J , Qian T, Wang M et al. Molecularly imprinted polymer enables high-efficiency recognition and trapping lithium polysulfides for stable lithium sulfur battery. Nano Lett 2017; 17: 5064–70. 28691822 10.1021/acs.nanolett.7b02332

[30] Patel MU , Dominko R. Application of in operando UV/vis spectroscopy in lithium–sulfur batteries. ChemSusChem 2014; 7: 2167–75. 25044737 10.1002/cssc.201402215

[31] Ni X , Qian T, Yan C et al. High lithium ion conductivity LiF/GO solid electrolyte interphase inhibiting the shuttle of lithium polysulfides in long-life Li–S batteries. Adv Funct Mater 2018; 28: 1706513.

[32] Xu N , Qian T, Liu J et al. Greatly suppressed shuttle effect for improved lithium sulfur battery performance through short chain intermediates. Nano Lett 2016; 17: 538–43. 27977209 10.1021/acs.nanolett.6b04610

[33] Kim H , Lee J, Ahn H et al. Synthesis of three-dimensionally interconnected sulfur-rich polymers for cathode materials of high-rate lithium–sulfur batteries. Nat Commun 2015; 6: 7278. 26065407 10.1038/ncomms8278 PMC4490390

[34] Ghazi ZA , He X, Khattak AM et al. MoS_2_/celgard separator as efficient polysulfide barrier for long-life lithium–sulfur batteries. Adv Mater 2017; 29: 1606817. 10.1002/adma.201606817 28318064

[35] Chung SH , Manthiram A. High-performance Li−S batteries with an ultra-lightweight MWCNT coated separator. J Phys Chem Lett 2014; 5: 1978–83. 26273884 10.1021/jz5006913

[36] Su YS , Manthiram A. Lithium–sulphur batteries with a microporous carbon paper as a bifunctional interlayer. Nat Commun 2012; 3: 1166. 23132016 10.1038/ncomms2163

