# Supplementary material for: Single lithium-ion channel polymer binder for stabilizing sulfur cathodes
Source: Natl Sci Rev. 2019 Oct 12;7(2):315–23. doi: 10.1093/nsr/nwz149 (PMC8288923; doi:10.1093/nsr/nwz149)
Supplement: nwz149_Supplemental_File [file nwz149_supplemental_file.docx]

**Single Lithium–ion Channel Polymer Binder for Stabilizing Sulfur Cathodes**

Chaoqun Niu, Jie Liu, Tao Qian*, Xiaowei Shen, Jinqiu Zhou, Chenglin Yan*

College of Energy, Collaborative Innovation Center of Suzhou Nano Science and Technology, Key Laboratory of Advanced Carbon Materials and Wearable Energy Technologies of Jiangsu Province, Soochow University, Suzhou 215006, China.

Corresponding authors. E-mails: tqian@suda.edu.cn; c.yan@suda.edu.cn





Figure S1. Synthetic procedure of PHHP samples.


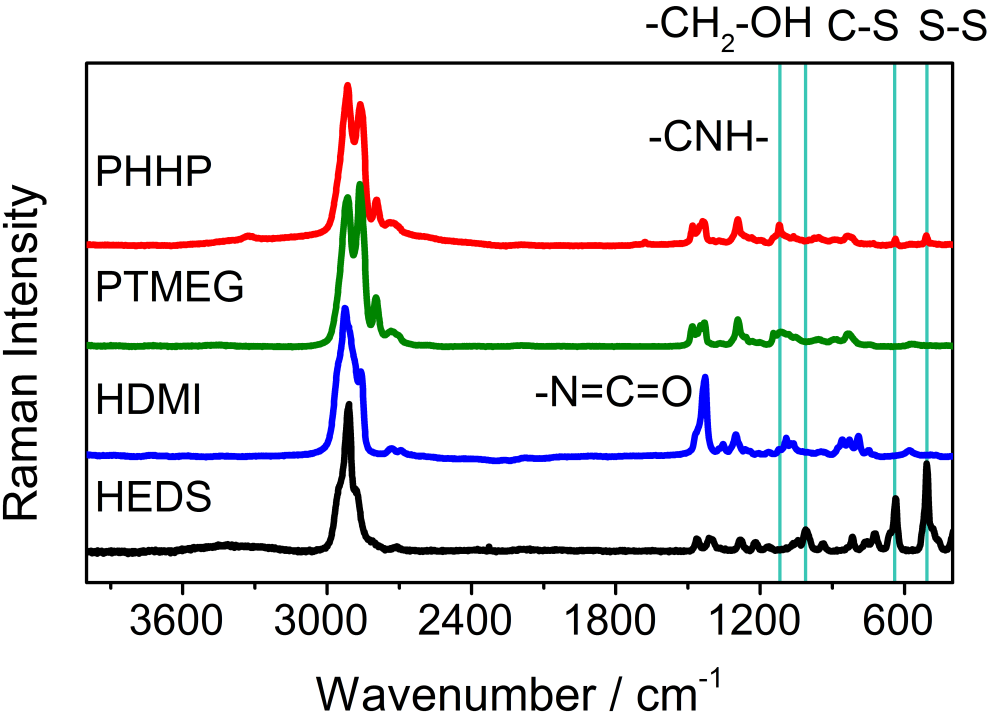


Figure S2. Raman spectra of HIDS, HDI, PTMEG and PHHP.


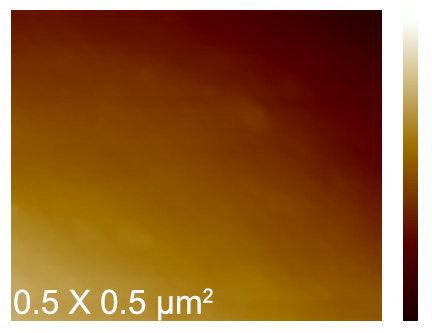


Figure S3. AFM image for PHHP film, color scale, 0 – 250 nm.
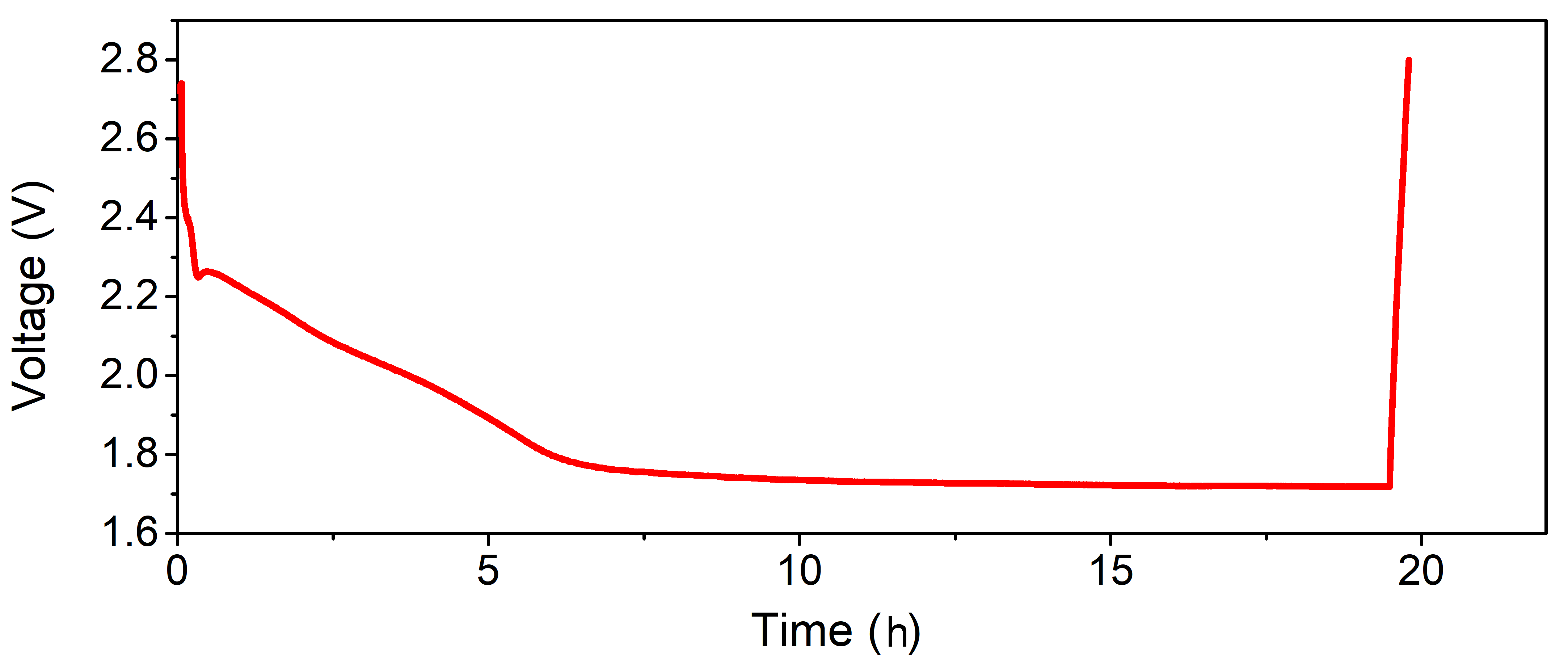


Figure S4. Discharge-charge curves of cell with PHHP electrode at 10 μA cm^–2^.


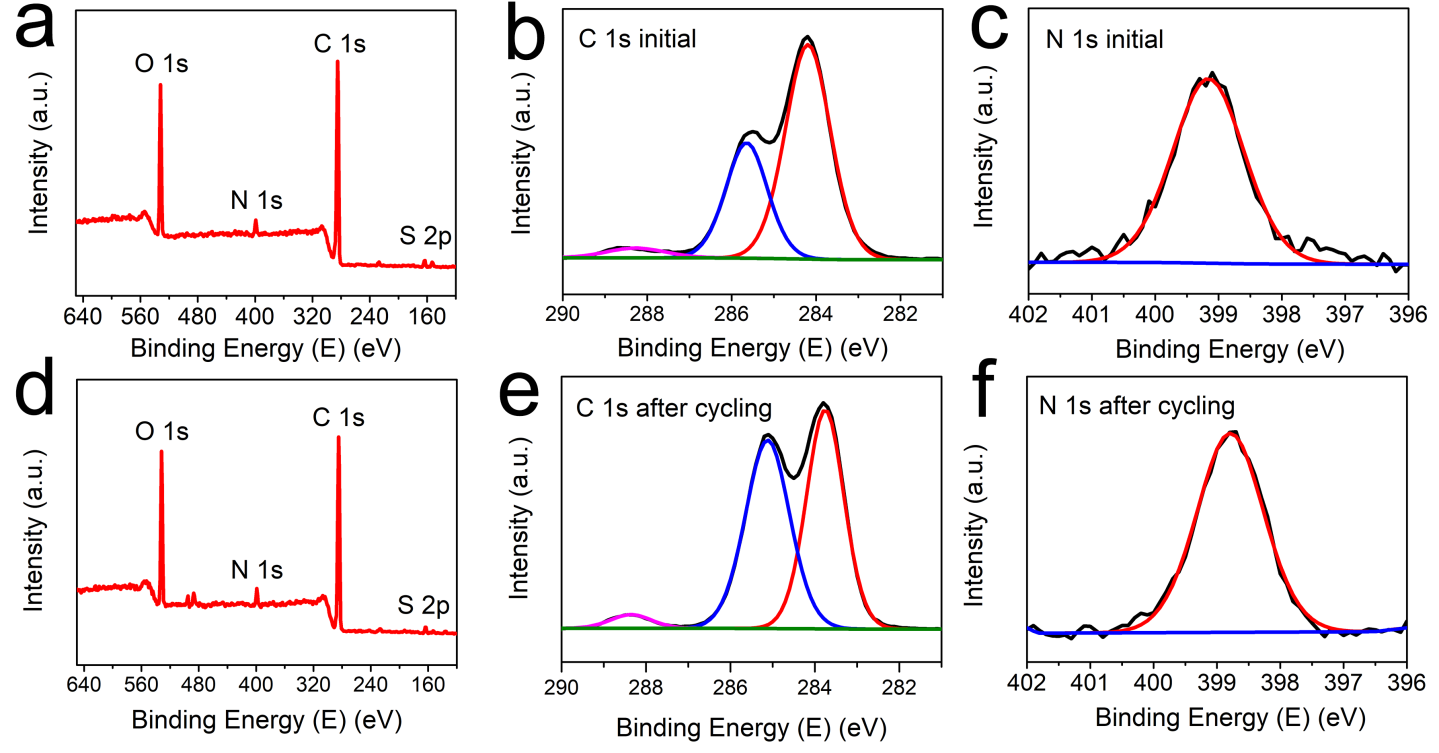


Figure S5. The XPS spectra of initial PHHP electrode and after cycling PHHP electrode. (a) XPS spectrum (b) C 1s spectra, (c) N 1s of initial PHHP electrode. (d) XPS spectrum, (e) C 1s spectra, (f) N 1s spectra of PHHP electrode after a discharge-charge cycle.


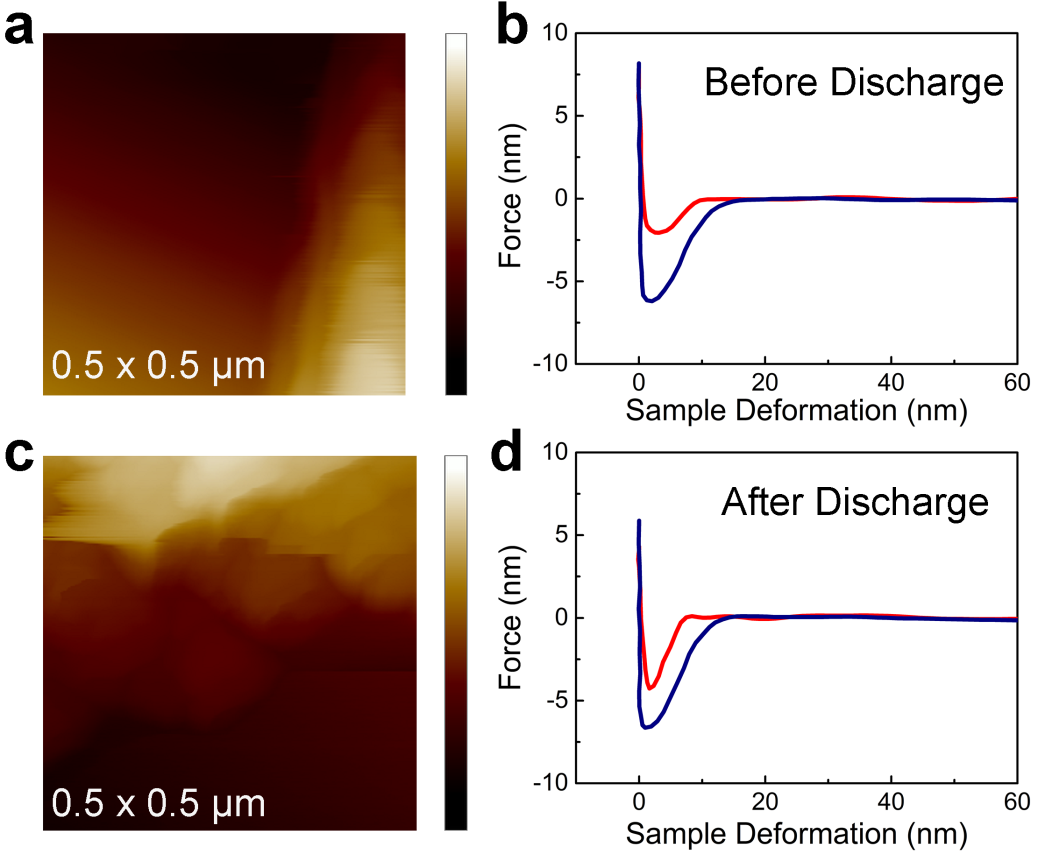


Figure S6. AFM image for (a) initial sulfur cathode and (c) discharged sulfur cathode with PVDF binder, color scale, 0 – 600 nm. Typical force–displacement curve of (b) initial sulfur cathode and (d) discharged sulfur cathode with PVDF binder.


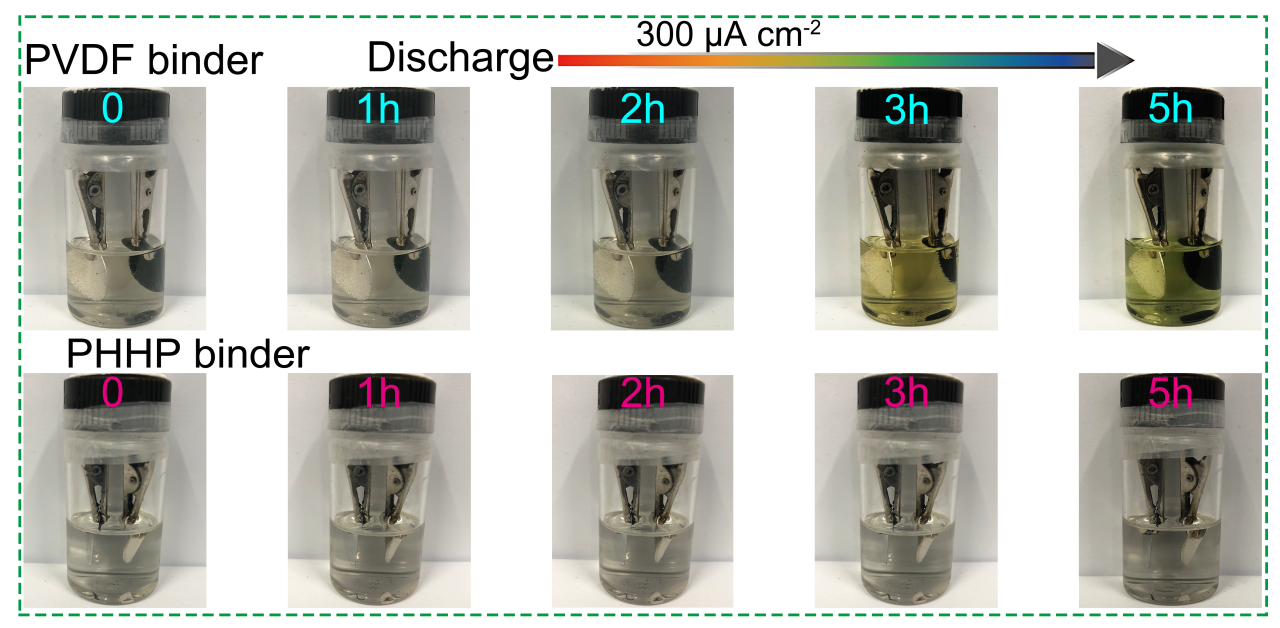


Figure S7. Photographs displaying the dissolution of polysulfide intermediates into electrolyte during discharge at 0.5 C for the Li-S with PVDF binder cell, contrary to the colourless electrolytes of the Li-S with PHHP binder.


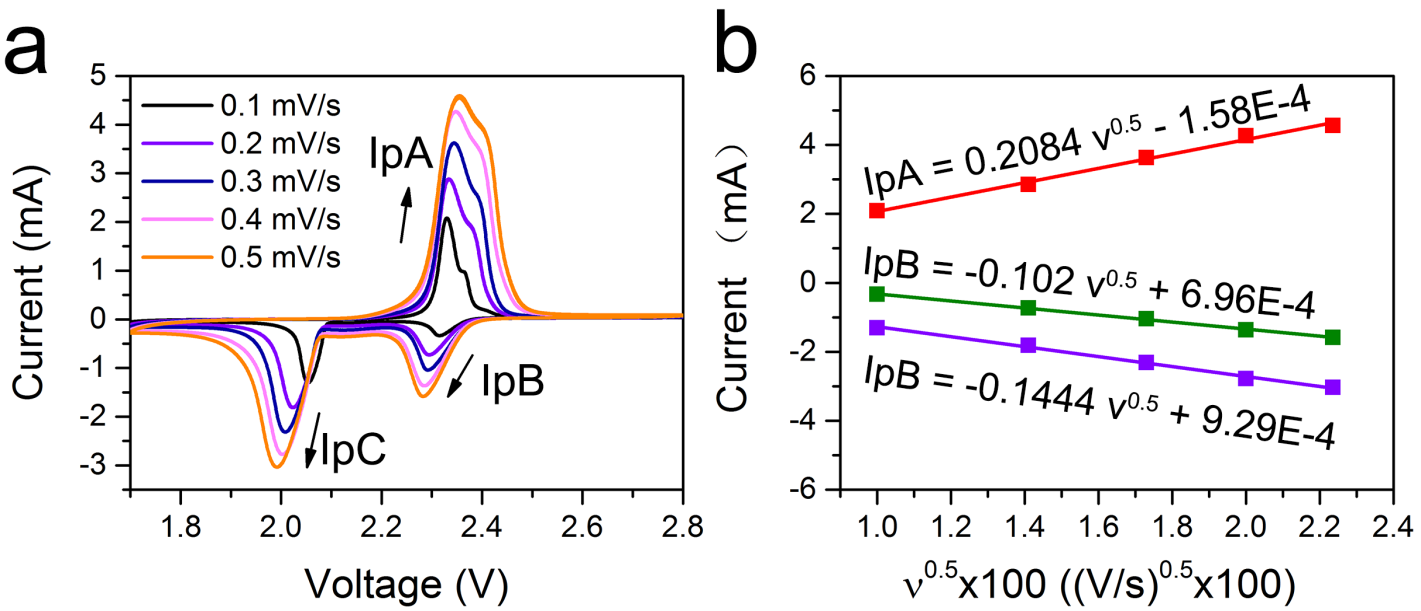


Figure S8. (a) Representative voltammograms of the sulfur cathode with PVDF binder obtained at different scan rates. (b) Linear relationship of I_p_ and v^0.5^.
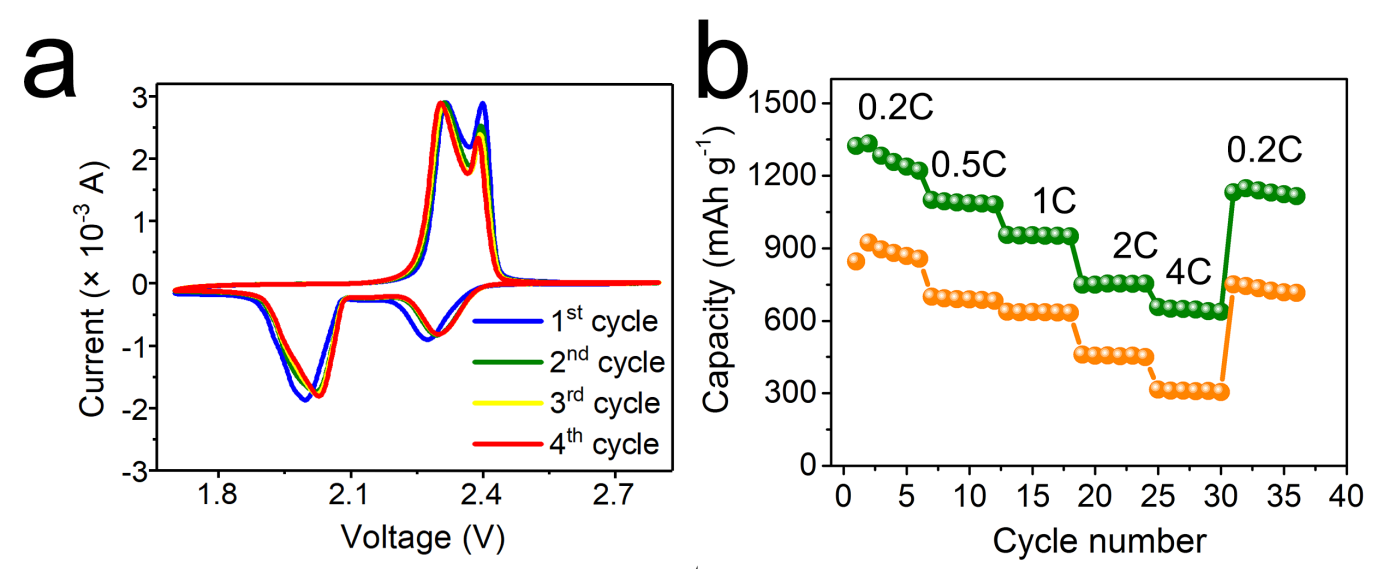


Figure S9. (a) CV profiles of the Li-S cells with PHHP binder at a scan of 0.1 mV s^-1^ in the potential range of 2.8 to 1.7 V. (b) Rate performance of Li-S cells with PHHP and PVDF.


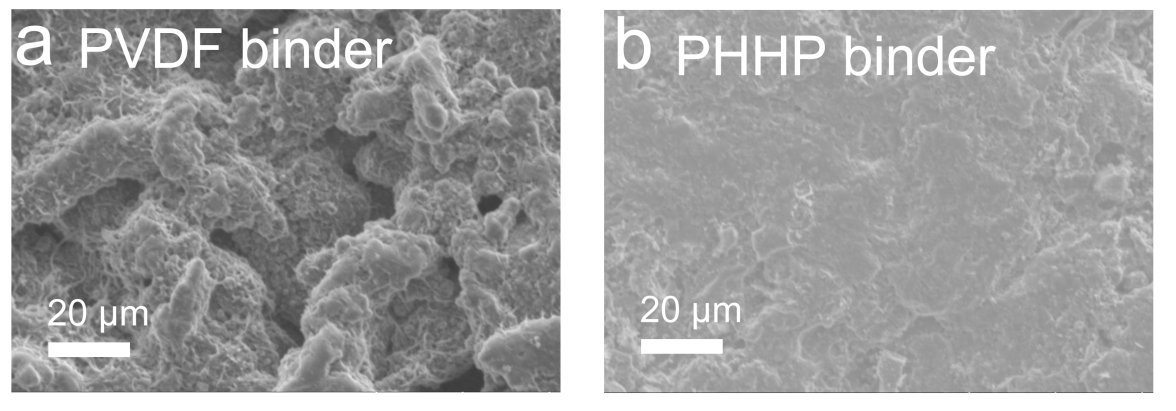


Figure S10. SEM images of (a) sulfur cathode with PVDF binder (b) sulfur cathode with PVDF binder after 50 cycles at current rate of 0.5 C.
